# The association between the genetic structures of commonly incompatible plasmids in Gram-negative bacteria, their distribution and the resistance genes

**DOI:** 10.3389/fcimb.2024.1472876

**Published:** 2024-11-26

**Authors:** Lei Fang, Ruyan Chen, Chenyu Li, Jingjing Sun, Ruishan Liu, Yanhao Shen, Xiaobing Guo

**Affiliations:** ^1^ Department of Laboratory Medicine, The First Affiliated Hospital of Zhengzhou University, Zhengzhou, China; ^2^ Collaborative Innovation Center for Diagnosis and Treatment of Infectious Diseases, State Key Laboratory for Diagnosis and Treatment of Infectious Diseases, The First Affiliated Hospital, Zhejiang University School of Medicine, Hangzhou, China

**Keywords:** genetic structures, plasmids, horizontal gene transfer, resistance genes, genetic element

## Abstract

Incompatible plasmids play a crucial role in the horizontal transfer of antibiotic resistance in bacteria, particularly in Gram-negative bacteria, and have thus attracted considerable attention in the field of microbiological research. In the 1970s, these plasmids, housing an array of resistance genes and genetic elements, were predominantly discovered. They exhibit a broad presence in diverse host bacteria, showcasing diversity in geographic distribution and the spectrum of antibiotic resistance genes. The complex genetic structure of plasmids further accelerates the accumulation of resistance genes in Gram-negative bacteria. This article offers a comprehensive review encompassing the discovery process, host distribution, geographic prevalence, carried resistance genes, and the genetic structure of different types incompatible plasmids, including IncA, IncC, IncF, IncL, IncM, IncH, and IncP. It serves as a valuable reference for enhancing our understanding of the role of these different types of plasmids in bacterial evolution and the dissemination of antibiotic resistance.

## 1 Introduction

In modern medicine and clinical practice, widely used antimicrobials and medications have significantly improved human health. However, the emergence of antibiotic resistance poses severe challenges to public health and clinical treatment (Di Pilato et al., 2024). Typically, *Escherichia coli* bacterial genetic material contains numerous resistance genes, which enable the host bacteria to express antibiotic resistance. Plasmids, as extrachromosomal genetic elements, can undergo horizontal transfer among bacterial strains. Therefore, resistance genes carried by plasmids play an essential role in the horizontal transfer of antibiotic resistance genes that cannot be ignored. In 1952, Joshua Lederberg first proposed the concept of plasmids, initiating discussions on the systematic classification of plasmid systems (Lederberg, 1952). García-Bartels et al. provided a comprehensive review of the history of plasmid classification. Major plasmid classification schemes include fi+ and fi- plasmid typing (based on conjugative transfer ability), superinfection immunity typing (based on exclusion phenomena), incompatibility plasmid replicon typing (based on replicons), MOB typing (based on relaxase genes), and classification schemes based on plasmid whole genome analysis (Garcillán-Barcia et al., 2023). In accordance with the PBRT typing method, incompatible plasmids are classified into 27 types (Villa and Carattoli, 2020). Given the widespread distribution and conjugative potential of IncA, IncC, IncF, IncL, IncM, IncH, and IncP plasmids in Gram-negative bacteria, this review offers a comprehensive analysis of their characteristics. Additionally, other types of incompatible plasmids (such as IncN, IncI, IncQ, IncX, etc.) are also crucial in microbial resistance. The presence of these plasmids reflects the diverse genetic strategies that bacteria adapt to the environment and antibiotic pressure. The resistance genes carried by these plasmids are closely related to the origin and ecological environment of the strains, playing crucial roles in the transmission and evolution of resistance (Tobin et al., 2024). However, as plasmids spread, the carried resistance genes also show a gradual evolution. Under different time and environmental conditions, plasmids can capture new resistance genes, and they may also lose existing ones (Ghaly et al., 2022). This evolution not only reflects the continuous adaptation of bacteria to antibiotic pressure but also has profound implications for the evolution of resistance genes. Therefore, we need in-depth research on the evolutionary dynamics of these resistance genes to better understand the formation and development of bacterial resistance to antimicrobials.

## 2 The primary mechanisms of antibiotic resistance in bacteria

The detection rates of multidrug-resistant (MDR), extensively drug-resistant (XDR), and pandrug-resistant (PDR) strains are continuously increasing, closely associated with the overuse of antibiotics and the spread of resistance genes mediated by mobile genetic elements. Bacteria express antimicrobials resistance primarily through three major mechanisms. Firstly, they reduce the intracellular concentration of antibiotics. For example, Gram-negative bacteria decrease antibiotic permeability by producing biofilms and actively expel antimicrobials via efflux pumps such as OqxAB and TmexCD-ToprJ.Secondly, they alter antibiotic targets. Recent studies have found that mutations in the KPC-2 resistance gene, leading to KPC-33 or KPC-189, confer resistance to ceftazidime and avibactam (Zhang et al., 2024). Lastly, bacteria inactivate antimicrobials by producing hydrolytic enzymes (Blair et al., 2015). β-lactam antibiotics are widely used for infections caused by Gram-negative Enterobacteriaceae, thus resistance problems arising from β-lactamases produced by bacteria have garnered significant attention. β-lactamases are mainly classified into four classes: A, B, C, and D. Among them, serine β-lactamases are classified into three types: Class A (e.g., KPC, SHV, TEM, and CTX-M), Class C (e.g., AmpC), and Class D (e.g., OXA), while Class B refers to metallo-β-lactamases, with common examples including VIM, IMP, and NDM (Karampatakis et al., 2023). Furthermore, there is a growing trend in the resistance to aminoglycosides, phosphomycin, and polymyxins (Luo et al., 2024). The resistance mechanism of aminoglycosides is primarily associated with bacterial production of 16S rRNA methyltransferases and is often clinically managed by combination therapy with β-lactam antibiotics to control infections (Yang and Hu, 2022). The resistance mechanism of phosphomycin is mainly related to defects in transport proteins, modifications of targets, and inactivation mediated by *fosA (* Huang et al., 2021). Polymyxins are often considered the last line of defense against infections; however, the increasing resistance rate is a cause for significant concern. Its resistance is associated with multiple genes, including chromosomal genes *pmr*CAB, *pho*PQ, *mgr*B, *pmr*D mutations, and plasmid-borne mcr genes (Luo et al., 2017). Mobile genetic elements such as plasmids, transposons, and insertion elements accelerate the spread of β-lactamase resistance genes along this transmission chain, posing a significant threat to human health. Furthermore, there is an urgent need for systematically developed and regularly updated antibiotic treatment regimens tailored to different resistant bacteria.

## 3 Bacterial mobile genetic elements

It is worth noting that although different groups of incompatible plasmids have differences in strain origin and gene carriage, they share many similar genetic elements, such as insertion sequences, transposons, and integrons. Insertion sequences (IS), transposons, and integrons are discrete DNA segments that can transfer themselves or even carry surrounding resistance genes to other locations in the genome through a cut-and-paste mechanism. Initially, the main distinction between insertion sequences and transposons was thought to be the presence or absence of passenger genes, but as more gene elements have been detected, the boundaries between them have become increasingly blurred (Siguier et al., 2014). IS elements are primarily classified into 17 families based on their structural characteristics, including IS*1*, IS*3*, IS*4*, IS*5*, IS*6*, IS*21*, IS*30*, IS*66*, IS*91*, IS*110*, IS*200*/*605*, IS*256*, IS*630*, IS*982*, IS*1380*, ISAS*1*, and ISL*3*. Jacques Mahillon and Michael Chandler provided a comprehensive review of these families (Mahillon and Chandler, 1998). Two transposon families mainly associated with antibiotic resistance are Tn*3* and Tn*7-like* transposons (Partridge et al., 2018). ISfinder (www-is.biotoul.fr) is a dedicated database for querying and annotating IS elements (Siguier et al., 2006). Furthermore, transposases can be classified into four types based on their structural features: DD(E/D) transposases, HUH single-strand DNA transposases, serine transposases, and tyrosine transposases. Integrons consist of three parts: the gene intl encoding the integrase, the gene attI encoding the recombination site, and the gene Pc encoding the promoter. Integrons are classified into types 1, 2, and 3 based on the similarity of their integrase genes, with type 1 integrons being frequently associated with microbial resistance (Bhat et al., 2023). These genetic elements not only help maintain the presence of resistance genes but also play an essential role in the horizontal transmission of plasmids. They facilitate plasmids in capturing exogenous genes and adapting to environmental changes, thereby promoting plasmid diversity and dissemination among different bacterial species. The presence of conjugative transfer regions in plasmids further enhances the spread of resistance genes. In these regions, there are regulatory factors and transfer genes that form a complex regulatory network.

## 4 The genetic structure of incompatible plasmids

**Figure 1:**
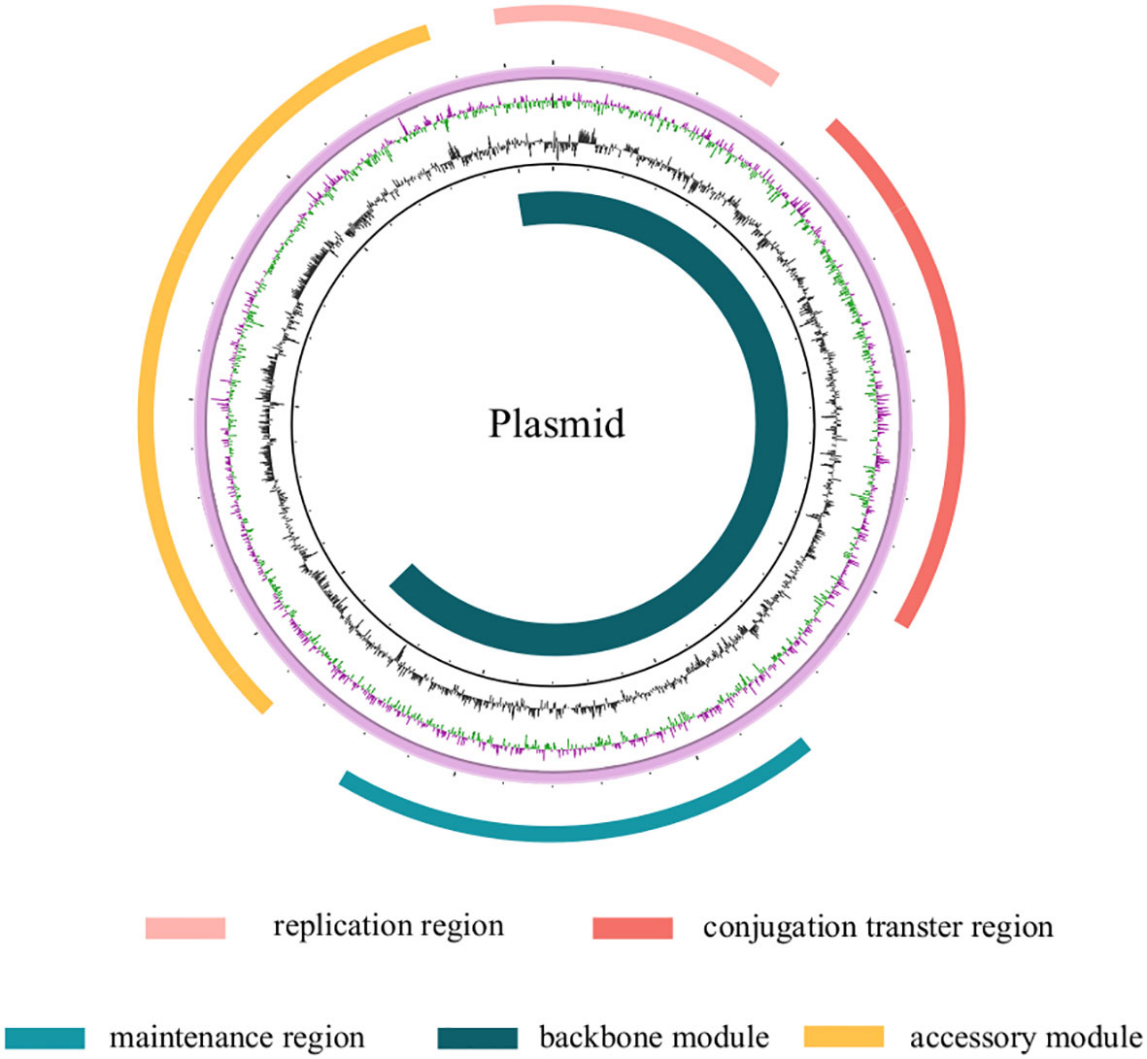
The plasmid genome is composed of two parts: the backbone module and the accessory module. The backbone module includes a replication region, a stability maintenance region, and a conjugation transfer region, while the accessory module is mainly comprised of antibiotic resistant genes. The two modules are not distinct from each other, but rather, they are interspersed.

Describing the biological characteristics of plasmids, which are crucial structures for bacterial resistance, is particularly important. Currently, plasmids of interest in Gram-negative bacteria are classified into 27 incompatibility groups, including IncA/C, B, F, H, I, L/M, K, P, T, O, X, W, R, Y, and their variants (Chen et al., 2024). These plasmids vary in size from several thousand to tens of thousands of base pairs (see **Table 1** for the sizes of plasmids described in this article). They are extrachromosomal genetic material independent of the chromosome, consisting primarily of core and accessory genomes. The core genome refers to shared genomic sequences among all plasmids of this type, typically encompassing replication region, the maintenance region, and the conjugation transfer region. The replication region of plasmids contains *rep* genes, which control plasmid DNA synthesis and facilitate vertical transmission. Research also suggests that mutations in the antisense stem-loop RNA structure (RNAI) of incompatible plasmids affect the translation of rep mRNA, thereby altering plasmid compatibility (Rozwandowicz et al., 2023). Additionally, the conjugative transfer region contains *tra* genes, enabling horizontal transfer via the concerted action of the type IV secretion system (*T4SS*) and relaxase. The accessory genome comprises bacterial genome variable regions, primarily containing insertion sequences, transposases, integrons, antibiotic resistance determinant clusters, and heavy metal detoxification proteins. Through these elements, plasmids can capture new genes, and even mutations, endowing host bacteria with additional traits, thus enabling bacterial survival in more complex environments. **Figure 1** displays the structural pattern of the plasmid. Mobile elements play a crucial driving role in the process of gene horizontal transfer. Insertion sequences, as part of transposons, act together with surrounding resistance genes to undergo horizontal transfer through a cut-and-paste mechanism, diversifying the genetic environment of resistance genes. This adaptation allows their hosts to cope with environmental challenges, potentially leading to the epidemic spread of a particular genotype of bacteria and the subsequent cross-species transmission (Macesic et al., 2023). Therefore, through in-depth research on the host distribution and geographical distribution of incompatible plasmids, conjugative transfer, carried resistance genes, and the function of genetic elements, we can have a more comprehensive understanding of the evolution process of bacterial resistance and provide more scientific guidance for tackling the problem of bacterial resistance.

**Table 1:** Characteristics of plasmids and the commonly carried antibiotic resistance genes.

Inc groups	Subtype	Size (bp)	β-lactamase resistance gene
IncA	A	52637- 272299	*bla*_VIM-1_, *bla*_SHV-12_
IncC	C	46056- 436531	*bla_TEM-1B_*, *bla*_CMY-2_, *bla*_NDM-1_, *bla*_CMY-4_, *bla*_CMY-6_ *, bla*_CMY-7_, *bla*_OXA-10_
IncF	FII: FIA: FIB	2592- 583215	*bla*_CTX-M-15_, *bla*_CTX-M-14_, *bla*_CTX-M-27_, *bla*_KPC-2_, *bla*_SHV-12_
IncH	HI, HII	50273- 477340	*bla*_CTX-M-9_, *bla*_TEM-1_ *, bla*_SHV-12_
IncL	L	26406- 167203	*bla*_OXA-48_, *bla*_CTX-M-9_ *, bla*_CTX-M-17_
IncM	M1, M2	29895- 156910	*bla*_CTX-M-4_, *bla*_OXA-48_ *, bla*_CTX-M-9_ *, bla*_IMP-4_
IncP	P-α、-β、-γ、-δ、-ϵ、-ζ	8187- 496601	*bla*_KPC-2_ *, bla*_OXA-2_

## 5 Distribution and association of incompatible plasmids with antibiotic resistance genes

### 5.1 IncA and IncC plasmid

The earliest occurrence of IncA and IncC plasmids may be associated with the use of antibiotics in animal husbandry (Johnson and Lang, 2012). The initial IncA plasmid was isolated from the fish pathogen *Aeromonas hydrophila* by Aoki et al. in 1971 and named pRA1 (accession number: FJ705807), later becoming the reference plasmid for IncA. The IncC plasmid was first discovered in the 1960s in *Salmonella typhimurium*, *Pseudomonas aeruginosa* (pIP40a), and *Klebsiella pneumoniae*, isolated from hospitals in Paris (Harmer and Hall, 2015). The historically significant IncC plasmid among them is the pR55, which was isolated from a strain of *Klebsiella pneumoniae* in 1969 and is also the oldest known IncC plasmid. The difference between IncA and IncC plasmids is the presence of a 69-nucleotide difference in the *repA* amplification sub-sequence, but they still share at least 98% identity. The genes necessary to perform replication (*repA*), conjugative transfer (*tra*), and plasmid partitioning (*stb* and *par*) make up the pRA1 plasmid’s backbone. The main difference between the pR55 and pRA1 plasmids lies in their accessory genes. The pR55 plasmid carries Tn*6178*, which contains the *catA1*, *addB, bla*_OXA-21_, *and sul1* resistance genes, as well as the ISCR*2* genetic element, which carries the *sul2* and *floR* resistance genes. On the other hand, the pRA1 plasmid carries the *sul2* and *tet* resistance genes (Doublet et al., 2012). Most IncC plasmids share a high degree of homology in their core genes, which include the essential genes for plasmid replication, distribution, stable maintenance, and conjugative transfer. In the early 1990s, Datta and Hedges, along with others, discovered that pRA1 (the reference plasmid for IncA) is compatible with plasmids from all known compatibility groups, including IncC plasmids. However, in 1972, Hedges observed that the pRA1 plasmid had significant entry exclusion from the IncC plasmid, so the IncA and IncC plasmids were called the “A-C complex”. IncA/C was later introduced and became popular. Ambrose et al. advised doing away with the labels IncA/C, IncA/C_1_, and IncA/C_2_ after demonstrating the compatibility of IncA and IncC replicons in 2018. They recommended referring to IncA/C as IncA and IncC individually or using “A/C,” “*repA/C*,” or “A-C complex” instead (Harmer et al., 2017; Ambrose et al., 2018). Specifically, IncA corresponds to IncA/C_1_, IncC to IncA/C_2_, and IncC comprises two types, C_1_ and C_2_. The primary distinction between C_1_ and C_2_ plasmids is that C_1_ is composed of *orf1832* and *rhs1*, whereas C_2_ includes *orf1847* and *rhs2* along with two insertion sequences i1 and i2 (Harmer and Hall, 2015). Mi-Jung Kim and colleagues conducted sequencing and analysis of A/C plasmids extracted from *photobacterium damselae subsp. piscicida* strains in the United States (pP91278) and Japan (pP99-018) during the 1990s. They found that compared to the pP99-018 plasmid, the pP91278 plasmid had an additional 1kb resistance determinant cluster and a conjugative transfer region IV (TRA IV). The remaining regions of the two plasmids showed >90% identity, providing favorable evidence for horizontal transfer of plasmids between bacteria and further evolution (Kim et al., 2008).

**Figure 2:**
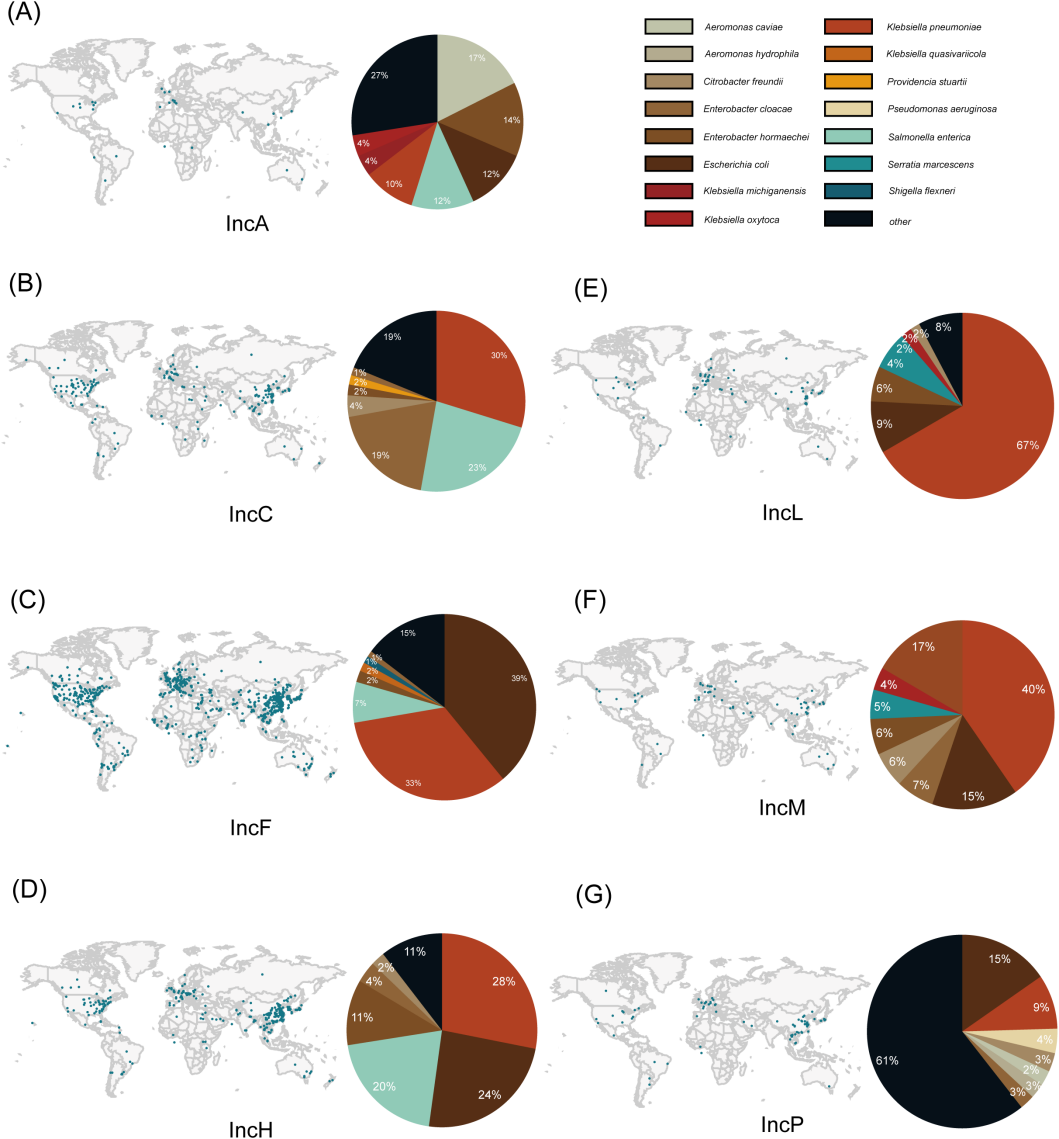
Global distribution of plasmids and host bacterial distribution. The global distribution map is obtained from the PLSDB database (Galata et al., 2019; Schmartz et al., 2022). The pie chart represents the distribution of plasmid host bacteria, displaying the top six most abundant plasmid types, with the remaining grouped as “other”. **(A–G)** represent the geographic distribution and host bacterial distribution of IncA, IncC, IncF, IncH, IncL, IncM, and IncP plasmids, respectively.

The IncA plasmid has been reported relatively infrequently, with a total of 51 cases distributed across the USA, Italy, China, Australia, the United Kingdom, Spain, Brazil, and other countries (**Figure 2A**). In contrast, the IncC plasmid has been reported more frequently, with a total of 831 cases distributed across 49 countries including the USA, China, India, Japan, and Germany. Among these countries, the United States and China have reported the highest number of IncC plasmids (**Table 2**). It has been found that IncA plasmids are mainly present in the host bacteria *Citrobacter freundii* (n=9 cases) (**Figure 2A**), while IncC plasmids are primarily distributed in bacteria such as *Klebsiella pneumoniae* (n = 247), *Salmonella enterica* (n=192 cases), and *Escherichia coli* (n=161 cases) (**Supplementary Material 1**) (**Figure 2B**). The specimens for isolation of IncA plasmids mainly comprise human perianal swabs, while IncC plasmids are predominantly sourced from human urine, blood, and perianal swabs. Additionally, isolation sources for both plasmids include strains from animals and the environment.

**Table 2:** Host bacterial information of plasmids and genetic environment of antibiotic resistance gene.

Inc groups	Total	Major host bacteria	Major country	Antibiotic-resistant genetic environment	Accession number
IncA	51	*Citrobacter freundii, Enterobacter hormaechei Escherichia coli*,	USA, Italy, China	–	–
IncC	831	*Klebsiella pneumoniae, Salmonella enterica Escherichia coli*,	USA, China, Japan	ISEcp*1-bla*_CMY-2_ *-bla-sugE*	HQ023863
					HQ023862
					FJ621588
				ISEcp*1-bla*_CMY-4_ *-blc-sugE*	AJ704863
				*sugE-blc-bla*_CMY-2_ *-ISEcp1*	KU160531
				*sugE-bla*_CMY-2-1_ *-bla*_CMY-2-1_ *-sugE*	CP000604
				ISEcp*1-bla*_CMY-6_	JN157804
IncF	10,778	*Escherichia coli, Klebsiella pneumoniae, Salmonella enterica*	China, USA, United Kingdom	*fosA3*-IS*26*-IS*26*-*bla*_TEM-1_-*bla*_CTX-M-3_-ISEcp*1*	JX627737
				*Tn3-like-ISEcp1-bla_CTX-M-15_-IS26-IS26*	GU371926
					GU371929
				*fosA3*-IS*26*-*intI1*-IS26-*bla*_TEM-1_-*bla*_CTX-M-3_-ISEcp*1*	JQ432559
				IS*1*-Tn*2*-IS*26*-intI*1*-dfr-qepA-ISCR*3*-groEL/intI1-rmtB-*bla*_TEM-1_-Tn*2*-IS*26*-Tn*2*-IS*26*-Tn*21*-IS*26*	JX997935
				IS*26*-*intl1*-*dfr*-*qepA*-ISCR*3*-*groEL/intI1*-*rmtB*-*bla*_TEM-1_-*ltnpR*-IS*26*	AB263754
					AM886293
					FJ183463
					FJ167861
IncH	1,618	*Klebsiella pneumoniae, Escherichia coli, Salmonella enterica*	China, USA, Japan	*IS26-bla*_SHV-12_ *-*IS*26*, IS*26-catA2-*IS*26*, IS*26-tetR(D)-tetA(D)-*IS*26*, IS*26-catA2-*IS*26*	KY270852
				IS*26-tetR(D)-tetA(D)-*IS*26*, IS*26-catA2-*IS*26*	KY270851
				*intI1-bla*_IMP-8_ *-aacA4-catB3-qacEdelta1-sul1-*ISCR*1-dfrA19-intl1-strA-strB*	EU855787
				*intI1-bla*_IMP-8_ *-aacA4-catB3-qacEdelta1-sul1-*ISCR*1-qnrB2-sul1-*ISCR*1-dfrA19-intl1-strA-strB*	EU855788
IncL	407	*Klebsiella pneumoniae, Escherichia coli, Enterobacter hormaechei*	Spain, Netherlands, China	*Tn2-bla*_TEM-1_ *-tnpA-res-frmB*	KM406489
				IS*1999-lysR-bla*_OXA-48_ *-*IS*1-*IS*1999*	KM406491
				IS*1999-*IS*1R-bla*_OXA-48_ *-lysR-*IS*1999*	KC335143
				IS*1999-lysR-bla*_OXA-48_ *-*IS*1999*	JN626286
IncM	179	*Klebsiella pneumoniae, Escherichia coli, Enterobacter cloacae*	USA, China Australia	*bla*_TEM-1_ *-tnpAcp2-aacC2-*IS*26-bla*_NDM-1_ *-trpF-bla*_DHA-1_ *-ampR-hypA-qac-sul1-*ISCR*1*	NC_019063
				*bla*_TEM-1_ *_-_* ISCfr*1-aacC2-IS26-bla*_NDM-1_ *-ampR-sul1-*ISCR*1-*ISEc*28-armA-*ISEc*29-msrE-mphE-*IS*26-*Tn*2*	NC_019889
				*int1-bla_IMP-1_-aac (6’)-IIc-qacL-qacE-delta1-sul1-istB-*IS*21*	AP024913
IncP	316	*Escherichia coli, Klebsiella pneumoniae, Pseudomonas aeruginosa*	China, Spain, Japan	aacA4-*bla*_IMP-9_-*aacA4*, *aacA4*-*catB8a*-*bla*_OXA-10_	KC543497
				*Intl1*-*aacA4*-*bla*_IMP-45_-*bla*_OXA-1_-*catB3*-*qacE1*-*sul1* (In*786*)	CP061377
				*Intl1*-*aacA4*-*bla*_IMP-45_-*guc35*-*bla*_OXA-1_-*catB3*-*qacED1*-*sul1*(In*786*)	KY883660

A/C-type plasmids, found in various geographical locations and host organisms, share a similar plasmid backbone: genes related to replication, maintenance, regulation, DNA metabolism, and conjugation transfer. Furthermore, the backbone includes many potential DNA-binding transcriptional regulation factors (referred to as nuclear-associated proteins, NAPs) that can reduce the fitness cost and expand the host range of the plasmid population. Accessory modules of A/C-type plasmids are mainly composed of insertion sequences, Transposases, class 1 integrons, antibiotic resistance genes, and heavy metal detoxification proteins genes (Rada et al., 2022). IncA and IncC plasmids exhibit high degree of gene homology and share highly conserved gene backbones. The A/C plasmid backbones region can be divided into the replication region (*repA*), stability region (*parAB*, *stbA*, and a toxin-antitoxin system), and conjugative transfer region (*aca*, *tra*). The prominent features of the backbones are the replication origin and Type IV secretion-like conjugative transfer system of A/C plasmids. The smallest sequenced IncC plasmid to date, pKPHS2, still carries the conjugative transfer gene *tra* and 13 antibiotic resistance determinants. The IncC plasmid backbone also contains the *dcm1*, *dcm2*, and *dcm3* genes, which encode DNA cytosine-5-methyltransferases and prevent degradation by host nucleases (Harmer and Hall, 2015; Ambrose et al., 2018). The reference plasmids for IncC type 1, pR148, and IncC type 2, pRMH760, have over 120 open reading frames in their backbone regions, encoding more than 100 amino acids (AAs). Toxin/antitoxin systems, DNA metabolism, conjugative transfer, partitioning, and stability, as well as several genes with unidentified roles, are among the genes necessary for replication (Del Castillo et al., 2013). The backbone regions of IncC plasmids of type 1 and type 2 differ by only 1%. This indicates that both types of plasmids have originated from a common ancestor and have undergone separate evolution in different lineages.

**Figure 3:**
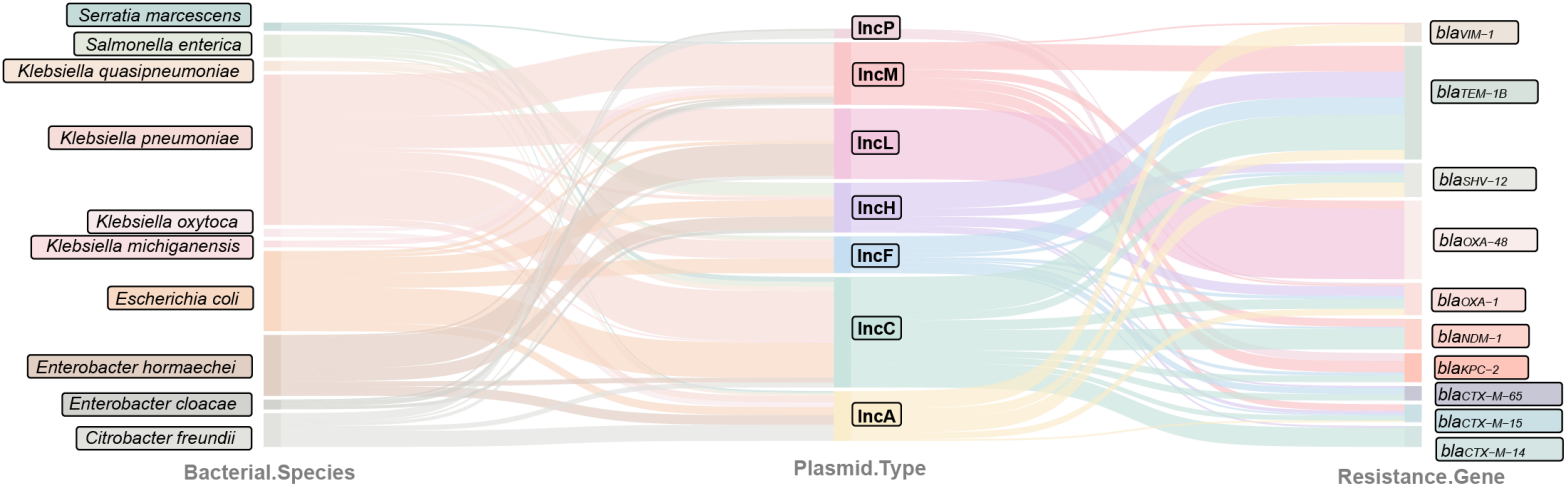
Relationships between plasmids, host bacteria, and β-lactam resistance genes. The data were sourced from the latest 50 discoveries of each plasmid type in the PLSDB database, with the top 10 host bacteria and genes by quantity used for the diagram.

**Figure 4:**
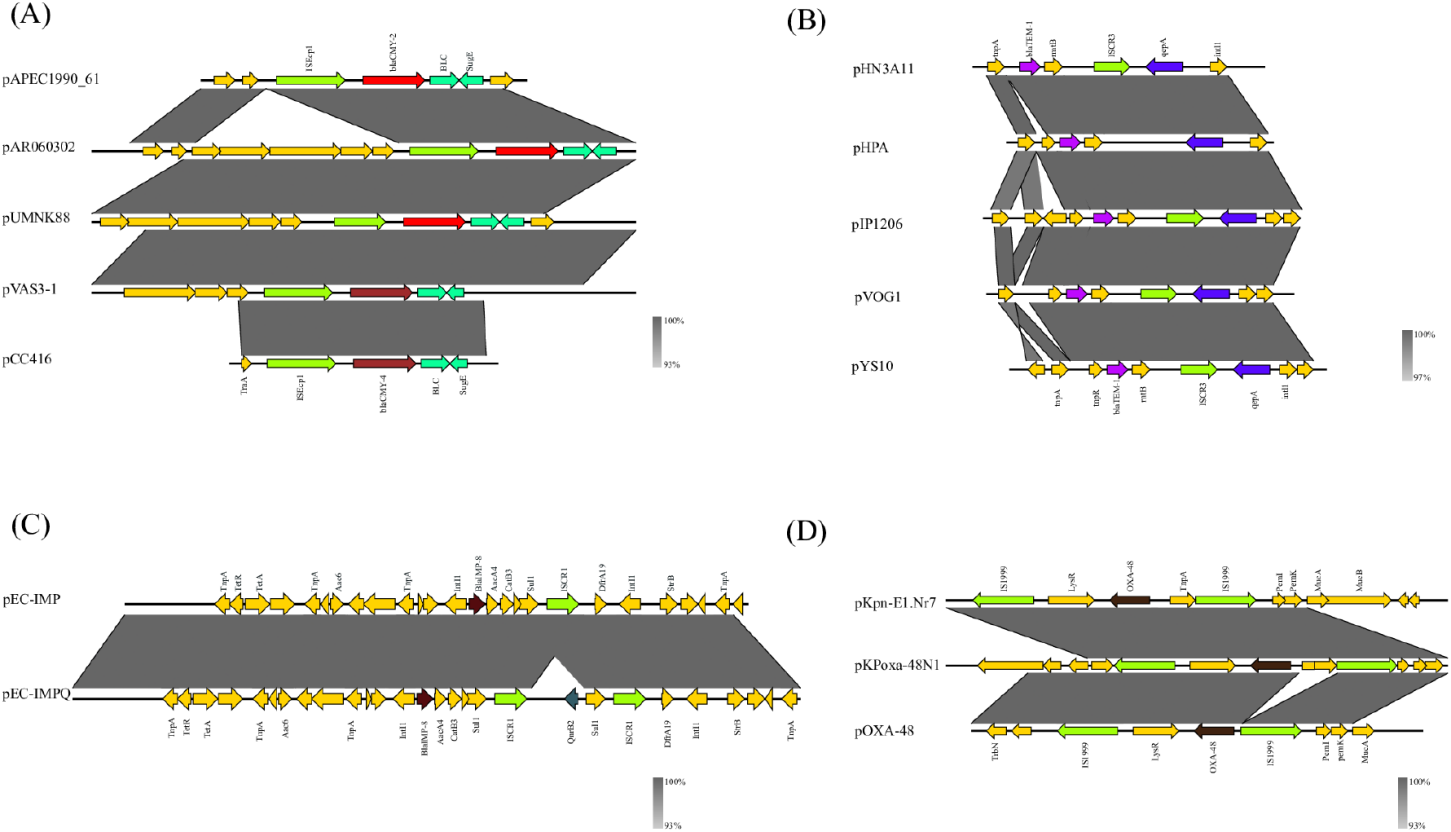
Genetic environment of plasmids carrying antibiotic resistance genes. **(A)** IncC plasmids: the genetic environment of blaCMY-2 in pAPEC1990_61, pAR060302, and pUMNK88, and of blaCMY-4 in pVAS3-1 and pCC416. **(B)** IncF plasmids: the genetic environment of blaTEM-1 in pHN3A11, pHPA, pIP1206, pVOG1, and pYS10. **(C)** IncH plasmids: the genetic environment of blaIMP in pEC-IMP and pEC-IMPQ plasmids. **(D)** IncL/M plasmids: the genetic environment of blaOXA-48 in pKpn-E1, Nr7, pKPoxa-48N1, and pOXA-48.

A/C plasmids are having a wide global distribution and commonly associated with multidrug resistances. Among them, IncA plasmids carry three potential resistance genes or clusters: *sul3* (sulfonamide resistance gene), *tetRA* (class D tetracycline resistance gene cluster), and *hipAB*-related gene clusters. Plasmid pRA1 exhibits resistance specifically to sulfonamides and tetracyclines (Fricke et al., 2009). The IncA plasmid primarily harbors β-lactam resistance genes, including *bla*_VIM_, *bla*_TEM-1B_, and *bla*_SHV-12_, and is known for its broad host range. Among IncC plasmids, the carriage rate of the carbapenem-coding resistance gene *bla*_NDM_ is the highest, with the *bla*_NDM-1_ subtype being particularly prevalent, which has emerged in IncC plasmids only recently. (**Figure 3**). Additionally, it is common to find the *bla*_CMY_ gene in IncC plasmids, with *bla*_CMY-2_ being the most predominant subtype, followed by *bla*_CMY-7_, and a small number of *bla*_CMY-4_ and *bla*_CMY-6_ subtypes. Additionally, the plasmids also carry carbapenem-coding resistance genes, primarily *bla*_NDM-1_, followed by *bla*_KPC-2_ and *bla*_IMP-4_. In research conducted by Christopher J. Harmer and colleagues, IncC plasmids containing both *bla*_NDM-1_ and *bla*_CMY-2_ are classified as Type 1, and all Type 1 IncC plasmids have ARI-A in the same location within their backbones. An array of antibiotic resistance genes can also be found in ARI-A, and both Type 1 and Type 2 IncC plasmids contain ARI-B. In IncC plasmids, the carriage rate of the carbapenem resistance gene *bla*_NDM_ is the highest, particularly with the prevalence of the *bla*_NDM-1_ subtype. This gene has only started to appear in IncC plasmids in recent years. Statistics show that almost all IncC plasmids carry the *sul1* or *sul2* gene, indicating inherent resistance to sulfonamide drugs. Plasmids containing the *bla*_CMY-2_ gene are usually surrounded by insertion sequences or transposons, of which plasmids pAR060302, pUMNK88_161, and p199061_160 share the same gene environment with ISEcp*1*-*bla*_CMY-2_-*blc*-*sugE* (**Figure 4A**) (**Supplementary Material 3**). Additionally, the plasmids pVAS3-1 carrying the *bla*_CMY-4_ gene and pCC416 share a genetic context identical to the aforementioned: ISEcp*1*-*bla*_CMY-4_-*blc*-*sugE* (**Table 2**). Furthermore, the plasmid pNDM-KN carrying the *bla*_CMY-6_ gene also contains ISEcp*1* genetic elements, with only one amino acid substitution (W661→L) compared to the *bla*_CMY-2_ gene (Carattoli et al., 2012; Partridge et al., 2018). The *bla*_CMY-2_ gene may potentially spread horizontally through the formation of a transposon unit (ISEcp*1*-*bla*_CMY-2_-*blc*-*sugE*), followed by recombination or mutation. In the process of interspecies transfer of resistance genes, the ISEcp*1* genetic element plays a crucial role. Relevant studies indicate that ISEcp*1* can provide a promoter for the captured gene at least, and it can mobilize adjacent DNA segments, which may explain the presence of *bla*_CMY-2_ and its variants in IncC plasmids. IncC carries resistance genes including *bla*_TEM_, *bla*_SHV_, *bla*_CTX-M_, *bla*_DHA_, *bla*_OXA_, *bla*_IMP_, *sul1*, *sul2*, *aphA1*, *aadA*, *aadB*, *strA*, *strB*, *aacC*, *tetA*, *floR*, *catA1*, and *dfrA*. Furthermore, on IncA/C plasmids isolated from *Escherichia coli*, the genes *fosA3* and *bla*_SFO-1_ have been found (Zhao et al., 2015). There are differences in the resistance genes carried by Type 1 and Type 2 IncC plasmids. The *bla*_TEM-1_ gene is more likely to be present in Type 2 IncC plasmids, while the *bla*_CMY-59_ gene is mainly distributed in Type 1 (Zhang et al., 2022). In addition, certain A/C plasmids also contain resistance genes associated with quaternary ammonium compounds (*qacE* and *sugE*) and mercury-related resistance genes, such as pSH163_135 (accession number: JN983045) and pSH696_135 (accession number: JN983048) (Han et al., 2012).

Compared to mutations occurring within plasmids themselves, inter-strain plasmid transfer contributes more to the survival of bacterial populations. However, the transfer frequency is mainly influenced by factors such as recipient bacteria, donor bacteria, compatibility of resident plasmids, and experimental conditions for conjugation. Although transfer frequencies may be high, they do not necessarily confer a survival advantage to bacterial populations (Geoffroy and Uecker, 2023). A/C plasmids belong to transferable plasmids, but different A/C plasmids exhibit varying transfer frequencies. Under natural conditions, the transfer frequency of IncA plasmid pRA1 in solid culture is 10^-2^, while in broth it is 10^-4^, and the transfer frequency of IncC plasmids ranges from 10^-7^ to 10^-4^ in both solid and broth cultures (Harmer and Hall, 2015). In comparison, the transfer frequency of IncC plasmids is higher than that of IncF plasmids. Factors controlling the transfer ability of IncC plasmids appear to be related to the chromosome. Genes involved in the conjugative transfer mechanism of A/C plasmids are mainly *tra* genes and *mobI* genes (Carraro et al., 2014). The conjugation transfer genes in the A/C plasmid have been identified by matching the protein to the known Tra protein; the IncA and IncC groups also have analogous genes. The transfer genes are divided into two distinct regions. The first region contains three sets of genes, which were isolated through seven unknown functional reads. The first group consists of *traI* and *traD*, the second group consists of *traL*, *traE*, *traK*, *traB*, *traV*, and *traA*, and the third group consists of *traC*, *trhF*, *traW*, *traU*, and *traN*. The final three transfer genes—*traF*, *traH*, and *traG*—are located in the second region. These genes produce mating pair stabilizing proteins, relaxation enzymes from the MOB_H_ group, coupling proteins, and proteins from the Mating pair formation (MPF) group that are essential for the assembly of the Type IV secretory system (T4SS), among other proteins involved in the conjugative processes. *AcaCD*, a transcription activator complex, is essential for the conjugative transfer of IncC plasmids. Its binding location is upstream of *traI*, *traL*, *traV*, *traN*, and *traF*, and it promotes the transcription of the tra genes. In addition, *AcaCD* also boosts the expression of 14 more IncC backbone areas, and potential *AcaCD* binding sites have been found in each of these regions (Gordils-Valentin et al., 2024). Therefore, its conjugative transfer frequency is influenced by multiple genes.

The IncA plasmid and the IncC plasmid are relatively small, and both can undergo conjugative transfer. However, the IncC plasmid is more abundant than the IncA plasmid, which may be related to the broader host range of IncC. The most common host for the IncC plasmid is *Klebsiella pneumoniae*, which has a more widespread distribution in nature. Currently, carbapenems are commonly used to treat infections caused by Gram-negative bacteria. The gene encoding carbapenemase, *bla*_VIM_, is frequently carried by the IncA plasmid, which raises the need for heightened vigilance.

### 5.2 IncF plasmid

The IncF plasmid was the first antibiotic resistance plasmid discovered in bacteria and is currently the most reported plasmid type. This plasmid can carry multiple replicons simultaneously, defining the FII, FIA, and FIB replicons based on the differences in copA, repA, and repB gene sequences. In 1960, Rintaro Nakaya and his colleagues isolated an IncF plasmid from *Shigella flexneri* in Japan, which belonged to the FII replicon group. Two years later, Yoshinobu Sugino and Yukinori Hirota proposed the association of this plasmid with antibiotic resistance (Sugino and Hirota, 1962). Most of the IncFI plasmids were isolated from the 1970s. In 2008, Bruno Pe´richon and others identified the IncF plasmid pIP1206 in *Escherichia coli* in France and conducted whole-genome sequencing. This plasmid has four replication regions: *repFIA*, *repFIB*, *repFIIA*, and *repFIIB*. The *repFIA* replication region of the pIP1206 plasmid is 99% identical to that of the pRSB107 plasmid. At the same time, the *repFIB* replication region shows a 99% consistency with the replication regions of both pRSB107 and pAPEC-O1-ColBM plasmids. The authors also proposed the viewpoint that pIP1206 is likely a new plasmid formed by recombination of pRSB107 and a pAPEC-O1-ColBM-like plasmid (Périchon et al., 2008). This indicates that plasmids can undergo recombination, similar to genes, and form new plasmids when their replicons are identical. This phenomenon allows for the bundling of resistance genes carried by plasmids, resulting in multiple resistance genes appearing on the same plasmid, thus conferring a higher level of drug resistance to the host bacteria. In 2010, it was first reported that an IncF plasmid carrying *bla*_TEM-1_, *bla*_SHV-12_, and *bla*_CTX-M-15_ genes was isolated from *Klebsiella pneumoniae* strains, which facilitated the simultaneous dissemination of these resistance genes (García-Fernández et al., 2010).

A total of 10,778 cases of IncF plasmids have been reported, with the highest prevalence found in *Escherichia coli* (n=4,287 cases), followed by *Klebsiella pneumoniae* (n=3,666 cases), *Salmonella enterica* (n=777 cases), *Enterobacter hormaechei* (n=221 cases), and *Klebsiella quasivariicola* (n=166 cases) (**Table 2**). IncF plasmids have been found in almost every country, with a prevalent distribution observed in countries such as China, USA, the United Kingdom, Spain, Japan and Australia (**Supplementary Material 1**) (**Figure 2C**). Samples from a diverse range of hosts have been identified as carriers of IncF plasmids, including humans, animals, and water sources. Within human samples, blood is the most common and significant source, followed by feces, urine, and sputum. Some IncF plasmids have also been found in abdominal drainage fluid, pus, bile, and perianal swabs.

The IncF plasmid is characterized by its large size and exhibits a complex mosaic structure. Among the multiple replicons mentioned above, the FII replicon is the most common type, followed by the FIB replicon. Additionally, IncF plasmids typically have the ability to carry multiple replicons, thereby expanding their host range (Ammar et al., 2024). Based on the typical replicons FII, FIA, and FIB, a replicon sequence typing (RST) scheme has been proposed, represented using the FAB formula. For example, F1:A1: B-, where F represents the IncFII replicon, A represents the IncFIA replicon, and B represents the IncFIB replicon. According to our data analysis, F2: A-: B- plasmids are the most prevalent, and the majority of them carry the antibiotic resistance gene *bla*_CTX-M-15_. Clearly, this plasmid formula has become the major carrier for spreading antibiotic resistance genes. The backbone region of IncF plasmids mainly includes replication-related genes (*repFIA*, *repFIB*, *repFII*, etc.), plasmid maintenance genes (toxin-antitoxin systems such as type I: *hok-sok*, type II: *relBE*, *mazEF*, *vapBC*, *ccdAB*, *parDE*, *higAB*, *HipBA*, and *Phd-Doc*), and conjugative transfer genes (*tra*, *trb*, *artA*, and *finO*). Additionally, certain IncF plasmids also carry exclusion genes (*traS* and *traT*) responsible for plasmid incompatibility (Oluwadare et al., 2020). The accessory module contains plasmid resistance genes, cargo genes (such as *ompP*, *ychA*, and *ychB*), and virulence genes (Al Mamun et al., 2021).

IncF plasmids are most commonly associated with β-lactamase resistance genes, the highest carriage rate of which is observed for *bla*_TEM-1B_, followed by *bla*_CTX-M_, *bla*_SHV_, and *bla*_KPC_(**Figure 3**). The subtypes of *bla*_CTX-M_ found on IncF plasmids mainly include *bla*_CTX-M-1_, *bla*_CTX-M-3_, *bla*_CTX-M-9_, *bla*_CTX-M-14_, *bla*_CTX-M-15_, *bla*_CTX-M-27_, and *bla*_CTX-M-55_. Among these, the most common subtype is *bla*_CTX-M-15_, which is consistent with the findings of Yasufumi Matsumura et al (Matsumura et al., 2013). The most commonly found carbapenemase-resistant gene is *bla*_KPC-2_. Interestingly, we observed that, although the resistance genes carried by IncF plasmids and IncC plasmids do not completely overlap, there are ISEcp*1* gene elements upstream or downstream of the carried resistance genes. For example, in plasmids pEC_B24, pEC_L8, and pEC_L46, the resistance gene *bla*_CTX-M-15_ is present, and the surrounding gene environment contains the ISEcp*1* element. Additionally, the sequence ISEcp*1*-*bla*_CTX-M-15_ on plasmids pEC_B24 and pEC_L46 is located on a *Tn3*-*like* transposon, specifically within the Tn*3-*like–ISEcp*1*-*bla*_CTX-M-15_-IS*26*-IS*26* sequence (**Supplementary Material 3**). IS*26* plays a crucial role in gene recombination (Vázquez et al., 2021). A similar structure exists in plasmids identified by Xiaojie Chen et al., including pHN3A11 (accession number: JX997935), pHPA (accession number: AB263754), pYS10 (accession number: FJ167861), pVOG1 (accession number: FJ183463), and pIP1206 (accession number: AM886293), where a conserved sequence of IS*26*-Δintl*1*-*dfr*-*qepA*-ISCR*3*-groE/Δintl*1*-rmt*B*-*bla*_TEM-1_-ItnpR-IS*26* is present (**Figure 4B**). This indicates the significant role of IS*26* and ISCR*3* should not be underestimated (Chen et al., 2014). The Tn*3-like* transposon, as a mature transposable element, facilitates the transfer of the *bla*_CTX-M-15_ resistance gene between plasmids or hosts, which could potentially explain its widespread presence in IncF plasmids. The fact that the ISEcp*1* element appears not just upstream or downstream of *bla*_CTX-M-15_ but also upstream of *bla*_CTX-M-14_ suggests that it plays a significant function in the neighborhood of resistance genes in general (Dhanji et al., 2011). Research indicates that within the same bacterial species, there is a high degree of identity in the mobile element regions carried by plasmids. According to Ling Wang’s study, the 18.9 kb IS*26* composite transposon carried by IncF plasmids in *Escherichia coli* shows an identity rate as high as 97.4%. Additionally, apart from IS*26* and ISEcp*1*, IncF plasmids are frequently associated with mobile genetic elements such as IS*1*, IS*6100*, IS*6*, IS*903*, ISKpn*6*, ISKpn*27*, Tn*3-like*, Tn*1712*, In*27*, and In*54* (Gancz et al., 2021; Hounmanou et al., 2021). Other types of resistance genes carried by IncF plasmids include: *bla*_NDM-1_, *bla*_NDM-5_, *bla*_NDM-7_, *bla*_NDM-11_, *bla*_KPC-3_, *bla*_KPC-8_, *bla*_OXA-1_, *bla*_OXA-3_, *bla*_OXA-48_, *bla*_OXA-181_, *bla*_CMY-2_, *bla*_TEM-1_, *bla*_GES-5_, *mcr-1*, *mcr-9*, *armA*, *arr-3*, *rmtB*, *ΔqacE*, *qeqA*, *qnrS*, *qnrB*, *aadA2*, *aadA5*, *dfrA7*, *catB4*, *aac(6’)-Ib-cr*, *aph(3’)-Ib*, *aph(6’)-1d*, *acc (3’)-IId*, *floR*, *mph(A)*, *sul1*, *sul2*, *strA-strB*, and heavy metal resistance genes such as copper and silver (Stephens et al., 2020; Conte et al., 2022; Liang et al., 2022; Turton et al., 2022). Furthermore, it has been observed that the resistance gene *aac (6’)-Ib-cr* frequently co-occurs with *bla*_CTX-M-15_ or *bla*_TEM-1_, and *bla*_NDM-5_ is commonly found together with *rmtB* on IncF plasmids, which will further exacerbate the situation of multidrug resistance. Over time, an increasing number of new subtypes of resistance genes have emerged in plasmids. Juliana Buck Dias et al. were the first to identify an IncF plasmid carrying the *bla*_CTX-M-24_ gene in *Escherichia coli*, and ShiKai Wu et al. also identified an IncF plasmid carrying the *bla*_OXA-1041_ gene. This resistance gene shows high similarity to *bla*_OXA-917_ and *bla*_OXA-427_ *(* Dias et al., 2022; Wu et al., 2023). It is evident that the resistance spectrum of IncF plasmids is becoming increasingly broad, which implies the existence of mutations, transfers, or acquisitions of resistance genes. It is worth delving deeper into the mechanisms behind this phenomenon in order to alleviate the problem of the lack of effective drugs in clinical settings due to resistance.

The IncF plasmid’s conjugation transfer function is mainly regulated by three genes: *traM*, *traJ*, and *traY*. *traM* is involved in DNA processing and controls its own promoter, *P_m_*. *traJ* is the activator for the *tra* gene, while *traY-X* helps regulate DNA metabolism, T4SS, and metastasis. *traM* activates *P_y_* to create *traY*, which then stimulates *P_m_* and further boosts the expression of *traJ*. This causes the expression of the transfer Operon in the IncF plasmid, which leads to the production of relaxants (*traD*, *traI*, *traM*, *traY*), conjugated pili (*traA*, *traQ*, *traX, traP*, *traE*, *traL*, *traC*, *traW*, *trbC*, *traF*, *trbB*), pairing stable proteins (*traN*, *traG*, *traU*), and T4SS core proteins (*traB*, *traK*, *traV*), thus facilitating conjugation (Bragagnolo et al., 2020). As the concentrations of *TraM* and *TraY* go up, they will impede *P_m_* and *P_y_*. *TraM*, *TraJ*, and *TraY* form a regulatory loop that is stimulated by the protein *SfrA* (*ArcA*) and *IHF*, which affect the promoter *P_y_* in the IncF plasmid. The negative regulator, Antisense RNA (*finP*), can suppress the translation of *traJ* mRNA. Moreover, conjugation is linked to the entry of *TraS* and *TraT*, whose expression is managed by *traJ*. When *TraS* binds to *TraG*, it hinders the conjugation transfer (Frost and Koraimann, 2010).

The IncF plasmid, as the most abundant plasmid type, is widely distributed among various bacterial strains, with *Klebsiella pneumoniae* being the most common. It carries a rich reservoir of resistance genes and is prevalent in many countries. Additionally, its more complex plasmid structure poses a significant threat to human health.

### 5.3 IncH plasmid

IncH plasmids fall into one of two categories: IncHI and IncHII, with IncHI being the main type. Based on the repertoire of replication genes, IncHI plasmids can further be categorized into five different subtypes: IncHI1 through IncHI5 (Liang et al., 2017). In 1961, the IncHI plasmid (R27: accession number AF250878) was first identified in *Salmonella typhi* in the United Kingdom. This plasmid carries the *Tn10* transposon, which contains the tetracycline resistance gene. Similarly, in 1993, an IncHI plasmid (pHCM1) was isolated from *Salmonella typhi CT18*. The pHCM1 plasmid showed more than 99% sequence identity with the R27 plasmid and carries additional resistance determinants, including *dhfr1b*, *cat*, *mer*, *strAB*, *sul*II, and *bla*_TEM-1_ *(* Sherburne et al., 2000; Parkhill et al., 2001). In 1969, Antone A. Medeiros et al. discovered the prototype IncHI2 plasmid, R478 (accession number: BX664015), in *Serratia marcescens* in the United States. The complete nucleotide sequence of this plasmid was later sequenced by Matthew W. Gilmour et al. in 2004 (Gilmour et al., 2004). The R478 plasmid carries a cluster of resistance determinants including tetracycline (also located on the *Tn10* transposon), chloramphenicol, kanamycin, mercury, silver, copper, arsenic, and tellurite resistance genes. It is evident that the R478 and pHCM1 plasmids carry enriched resistance genes compared to the R27 plasmid. The R478 and pHCM1 plasmids may have captured additional resistance genes on the basis of the R27 plasmid (with the presence of insertion elements IS*26*, IS*1*, and the transposon Tn*10* providing more opportunities for plasmid-mediated acquisition of exogenous genes) and evolved from there. In 1985, E. Chaslus-Dancla et al. identified IncH plasmids in *Escherichia coli*. Subsequently, Timothy J. Johnson et al. first completed the sequencing of the IncHI2 plasmid (pAPEC-O1-R) isolated from *E. coli* in 2006, which shares a high similarity with the R478 plasmid (Chaslus-Dancla and Lafont, 1985; Johnson et al., 2006). IncHI2 plasmids were identified in a pneumonic *Klebsiella pneumoniae* (pK29) collected in 2001 and in two sewer *Escherichia coli* (pEC-IMP and pEC-IMPQ) collected in 2004 by Ying-Tsong Chen et al (Chen et al., 2007; Chen et al., 2009). Compared to plasmid R478, plasmid pK29 has two unique regions. One region contains the IS*26*-ISCR*1*-*bla*_CMY-8_-*qacEdelta1*-*sul1*-IS*26*-IS*6100* sequence, and the other region contains the *tnpA*-*bla*_CTX-M-3_-*tnpA* sequence. The main difference between plasmids pEC-IMP and pEC-IMPQ is the presence of the *qnrB2* resistance gene, which is also associated with different host resistance phenotypes (**Table 2**). The genetic environment of the resistance genes in pEC-IMP and pEC-IMPQ plasmids are “*intI1*-*bla*_IMP-8_-*aacA4*-*catB3*-*qacEdelta1*-*sul1*-ISCR*1*-*dfrA19*-*intl1*-*strA*-*strB*” and “*intI1*-*bla*_IMP-8_-*aacA4*-*catB3*-*qacEdelta1*-*sul1*-ISCR*1*-*qnrB2*-*sul1*-ISCR*1*-*dfrA19*-*intl1*-*strA*-*strB*” respectively (**Figure 4C**). It is evident that the upstream ISCR*2* gene element on *qnrB2* plays a significant role in the horizontal transmission of the *qnrB2* resistance gene.

A total of 1,618 cases of IncH plasmids have been identified, predominantly found in China, USA, the United Kingdom, Japan, Australia, Spain, South Korea, Canada, Switzerland and the Czech Republic. There are a total of 49 different host bacteria, with *Klebsiella pneumoniae* (n=454 cases), *Escherichia coli* (n=390 cases), and *Salmonella enterica* (n=329 cases) being the most commonly associated strains with this plasmid (**Supplementary Material 1**) (**Figure 2D**). The main isolation sources of the specimens are human blood, feces, sputum, and urine, while in animals, they are primarily isolated from cattle and chickens.

The backbone region of the IncH plasmid contains replicative genes (repHI1A, repHI1B, and repHI2 for IncHI plasmid replication, with five different subtypes, each containing specific replicative genes: IncHI1: *repHI1A*+*repHI1B*+*repFIA-like*, IncHI2: *repHI2A*+*repHI2C*, IncHI3: *repHI3B*+*repFIB-like*, IncHI4: *repHI4A*+*repHI4B*, and IncHI5: *repHI5B*+*repFIB-like*) *(* Liang et al., 2017). It also includes the partitioning modules (*Par1* and *Par2*), maintenance genes, and conjugative transfer genes (the *tra1* and *tra2* regions). Additionally, accessory modules contain resistance determinants and several mobile elements.

The IncH plasmid carries several β-lactamase genes, such as *bla*_TEM-1_, *bla*_SHV-12_, *bla*_CTX-M-1_, *bla*_CTX-M-8_, *bla*_CTX-M-9_, *bla*_SHV-12_, *bla*_IMP-8_, *bla*_NDM-1_, and *bla*_OXA-10_(**Supplementary Material 1**). Among the β-lactamase resistance genes, *bla*_CTX-M_ is widely observed, and its most common subtype is *bla*_CTX-M-9_. The *bla*_CTX-M-15_ subtype, which is known to have a complex genetic environment, was identified in an IncH plasmid named pENVA by Andreas Schlüter et al (Schlüter et al., 2014). The genetic environment surrounding *bla*_CTX-M-15_ consists of IS*26*-*aacC2*-*bla*_TEM-1_-*tnpRATn2*-ISEcp*1*-*bla*_CTX-M-15_-*tnp-*Tn*2*. *Bla*_TEM-1_ is located on the transposon Tn*2*, while *bla*_CTX-M-15_ is located on the transposon Tn*2012*. Interestingly, upstream of *bla*_CTX-M-15_, there exists a genetic element called ISEcp*1*, which is likely responsible for mobilizing the *bla*_CTX-M-15_ gene and integrating it into the *Tn2* transposon that contains *bla*_TEM-1_. In recent years, there has been a continuous increase in IncH plasmids carrying the *bla*_CTX-M-15_ subtype. These IncH plasmids also carry other resistance genes, including *tet*, *dfrA5*, *sul1*, *sul2*, *strA*, *strB*, *qnrB*, *mcr-1*, *mcr-3*, *mcr-9*, *aacC2*, *aadA*, *aphA1ab*, *catA1*, as well as heavy metal resistance genes (mercury, copper, silver, arsenic, and tellurium), and others (Fukuda et al., 2022; Wang et al., 2024).

The transfer of IncHI is mainly directed and expressed by *tra1* and *tra2* regions. These regions contain genes that code for *oriT* transfer initiation, relaxation bodies (*traI*), conjugating proteins (*TraG*), and Mpf proteins (*TrhY*, *TrhR*, *TrhF*, *TrhH*, and *TrhG*) *(* Lawley et al., 2002). The *tra2* region (*trhA*, *trhL*, *trhE*, *trhK*, *trhB*, *trhV*, *trhC*, *trhP*, *trhW*, *trhU*, and *trhN*) is responsible for encoding the Mpf protein and synthesizing H pili. The *trhO*, *trhZ*, and *htdA* genes can control the rate of metastasis, with *trhO* and *trhZ* increasing the frequency of metastasis and *htdA* having the opposite effect. The Mating pair formation (Mpf) apparatus is composed of pili protein subunits and pili protein processing proteins, which are connected to recipient bacteria to transfer plasmid DNA (Lawley et al., 2003). Research also indicates that in *salmonella typhi*, the conjugative transfer of IncHI plasmids is influenced by temperature. Between 22-30°C, approximately 10-80% of recipient bacteria can acquire IncHI1 plasmids, whereas at 37°C, almost no recipient bacteria acquire the plasmid. This phenomenon is primarily related to two mechanisms: first, the expression of transfer genes is inhibited or the transfer genes are unstable, and second, at 37°C, the Mpf apparatus becomes inactive. The temperature sensitivity of IncHI1 plasmid transfer confers an advantage for the dissemination of antibiotic resistance genes in environmental pathogens (Taylor, 2009).

Similarly, the IncH plasmid is also a broad-host-range plasmid, and it is relatively large. Clinically, colistin has been reintroduced for the treatment of infections caused by Gram-negative bacteria and is often used as a last-resort antibiotic. Genes associated with colistin resistance, such as *mcr-1* and *mcr-9*, are frequently linked to the IncH plasmid (Saavedra et al., 2017; Osei Sekyere et al., 2020). Moreover, this plasmid is associated with multiple β-lactam resistance genes and has been detected with the highest frequency in China, which may be related to the relatively lenient antibiotic usage policies in the country.

### 5.4 IncL plasmid and IncM plasmid

In 1970, Y. A. Chabbert and colleagues identified the IncM plasmid (R69) from *Salmonella paratyphi* and *Klebsiella pneumoniae* strains in France. This was the earliest discovery of such plasmids, and R69 carries the resistance genes *bla*_TEM-1_, *tetA(B)*, *aphA-1*, and *merA (* Chabbert et al., 1972). In 1973, Hedges and R. W. and their colleagues identified the IncL and IncM plasmids in *Escherichia coli* and classified them into separate groups. However, in 1978, Richards and Datta merged them and named them IncL/M (Hedges et al., 1974). Following the latest PBRT subtyping scheme proposed in 2015, Carattoli, A. et al. classified it into three different types: IncL, IncM1, and IncM2. In 1977, Richards, H. and Datta, N. identified the presence of an IncL plasmid (R471) in a strain of *Shigella flexneri* isolated from the United States. The plasmid was found to harbor the *bla*_TEM-1_ and *merA* resistance gene clusters (Carattoli et al., 2015). Although both R69 and R471 plasmids harbor the *bla*_TEM-1_ resistance gene, they have completely different genetic environments. Twenty-eight years later, Park, Yeon-Joon et al. also discovered IncL/M plasmids in *Shigella flexneri* strains in South Korea. However, the resistance genes carried by this type of plasmid are completely different from the resistance gene cluster in R471, including *bla*_CTX-M-3_, *bla*_CTX-M-12_, *bla*_OXA-1_, *aac (6’)-lb-cr*, and *armA*. During this time period, IncL/M plasmids may have undergone significant genetic evolution. Park, Yeon-Joon stated that the transfer rates of the plasmids carrying ISCR*1*, *aac (6’)-Ib-cr*, *armA*, and *bla*_OXA-1_ were 100%, and there is a close association between the horizontal dissemination of *bla*_CTX-M_ resistance genes and the ISEcp*1* genetic element (Park et al., 2009). Evidence once more affirms that ISCR*1* and ISEcp*1* are able to facilitate the horizontal dispersal of resistance genes by enabling the mobilization of resistance genes during conjugative transfer.

There are a total of 407 cases of IncL plasmids, with *Klebsiella pneumoniae* (n=273 cases) being the most common host bacterium (**Figure 2E**). These plasmids are primarily distributed in the Spain, Netherlands and China. IncM plasmids, on the other hand, have a total of 179 cases, with 94 cases of IncM1 and 85 cases of IncM2. The distribution of these two types of plasmids is relatively even. The most common host bacteria for them are *Klebsiella pneumoniae* (n=72 cases) and *Escherichia coli* (n=27 cases) (**Figure 2F**). Literature suggests that IncL/M plasmids mainly originate from *Enterobacteriaceae* in the Mediterranean region and Western Europe (Adamczuk et al., 2015). The primary isolation sources for the IncL plasmid in human specimens are urine, blood, and peritoneal drainage fluid. Additionally, besides these sources, the IncM plasmid is predominantly isolated from human perianal swab samples.

Although the backbones of IncL and IncM are different, they still share some common backbone region genes, including replication genes (*repA*), maintenance genes (*parA*, *parB*), the *tra* locus (*tra*), and some plasmid backbone stability genes including *pemI*, *pemK*, *mucA*, *mucB*, *resD*, and the transfer gene *trb*. Since plasmid pEL60 (accession number AY422214) does not carry any resistance determinant clusters or insertion sequences, it is considered to have the most typical IncL/M plasmid backbone genes. pEL60 only contains replication genes (*rep*), transfer genes (*trb*), stability genes (*pemI*, *pemK*, *parA*, *parB*), and the *tra* locus. In terms of maintenance genes, the importance of *parAB* genes is higher than that of *pemIK* genes (Mierzejewska et al., 2007). IncL and IncM plasmids both have exclusion genes (*excA*, *traY*) and relaxase genes (*traX*), although their sequences are not the same. Additionally, both IncL and IncM accessory modules include resistance genes and transposon elements.

There are still differences in the resistance genes present in the accessory modules of IncL and IncM plasmids. The β-lactam resistance gene predominantly carried by IncL plasmids is *bla*_OXA-48_, accounting for 88% of all IncL plasmids carrying resistance genes (**Figure 3**). Moreover, plasmids carrying *bla*_OXA-48_ alone are the most common among IncL/M plasmids, such as pRAYY (accession number: KX524525.1), pEC745 (accession number: CP015075.2), pKpn-E1. Nr7 (accession number: KM406491.1), and pKPoxa-48N1 (accession number: KC757416.2), all of which contain *bla*_OXA-48_ located on the transposon Tn*1999* (**Figure 4D**). The gene sequences upstream and downstream of Tn*1999* show a similarity of over 90%. Additionally, IncL/M plasmids isolated from patient samples by Linda Hadjadj and Nadim Cassir also carry Tn*1999*. This indicates that *bla*_OXA-48_ can stably exist on IncL plasmids and can be rapidly spread (Mierzejewska et al., 2007). In addition, other β-lactam resistance genes that co-occur with *bla*_OXA-48_ on the same plasmid include *bla*_CTX-M_. Other β-lactam resistance genes carried by IncL plasmids include *bla*_SHV_, *bla*_TEM_, *bla*_VIM_, and *bla*_KPC_. Other identified resistance genes include *sul1*, *merA*, and so on. We found differences in the β-lactam resistance genes carried by IncM1 and IncM2. *bla*_SHV_ and *bla*_NDM_ are only present in IncM1 plasmids, mainly the *bla*_SHV-30_ subtype. The most common β-lactam resistance gene in IncM1 plasmids is *bla*_OXA_, followed by *bla*_CTX-M_, while in IncM2 plasmids, the most common gene is *bla*_TEM_, followed by *bla*_IMP_. Other resistance genes carried by IncM1 and IncM2 include *tetA (AB)*, *aacAC*, *aadA*, *merA*, *sul1*, *armA*, *dfrA12*, *mph (AE)*, *qacG*, *qnrB2*, *catB3*, as well as resistance genes for heavy metals (mercury, cobalt, zinc, and cadmium) (Adamczuk et al., 2015). *Enterobacter cloacae*, *Klebsiella aerogenes*, and *Serratia marcescens* were recently isolated from a single patient specimen and were found to harbor the *bla*_IMP-1_ resistance determinant cluster on an IncL/M plasmid designated as pSL264. The *bla*_IMP-1_ resistance gene carried by this plasmid exhibits a distinctive genetic environment structure, which is depicted as *int1*-*bla*_IMP-1_-*aac (6’)-IIc*-*qacL*-*qacE*-*delta1*-*sul1*-*istB*-IS*21*. Furthermore, conjugation transfer experiments involving this plasmid have demonstrated the horizontal transmission of the resistance genes it carries (Mori et al., 2021). Additionally, a clinical strain of *Klebsiella pneumoniae* yielded an IncL/M plasmid termed pCTX-M360, which carries the *bla*_CTX-M-3_ gene. pCTX-M360, in contrast to pEL60 devoid of resistance genes or transposon elements, exhibits variations solely attributed to the presence of the ISEcp*1* gene element and Tn*2* transposon harboring the *bla*_CTX-M-3_ gene. Therefore, it can be inferred that the emergence of the pCTX-M360 plasmid involved multiple insertion events rooted in the pEL60 plasmid (Zhu et al., 2009). Plasmids not only act as carriers for transferring between bacterial species, but they also consistently acquire exogenous gene elements, thereby imparting additional characteristics to the plasmid.

The conjugative transfer genes of the IncL/M plasmid include *trbA*, *trbB*, *trbC*, *nikA*, *mobA*, *traH*, *traI*, *traJ*, *traM*, *traN*, *traO*, *traQ*, *traU*, *traW*, and *traY*, but their specific functions are still unclear. The expression of the *tir* gene in the IncL plasmid inhibits conjugative transfer, reducing the frequency of bacterial conjugation (Potron et al., 2014).

The gene encoding carbapenemase, *bla*_OXA-48_, is primarily located on the IncL plasmid, which shares a similar genetic background. Tn*1999* may play a crucial role in the dissemination of this resistance gene. In contrast, the IncM plasmid is mainly associated with the *bla*_VIM_ gene. The host bacteria for both plasmid types are predominantly *Klebsiella pneumoniae* and *Enterobacter hormaechei*, both of which are broad-host-range plasmids capable of conjugative transfer.

### 5.5 IncP plasmid

The IncP plasmid is called IncP-1 in the genus *Pseudomonas* and IncP in the family *Enterobacteriaceae (* Yakobson and Guiney, 1983; Adamczyk and Jagura-Burdzy, 2003). This plasmid can be classified into six subtypes: IncP-α, -β, -γ, -δ, -ϵ, and -ζ. However, as more IncP/IncP-1 plasmids are identified and typing methods become more refined, additional subtypes such as η, θ, ι, κ, o, λ, and μ have also emerged gradually (Popowska and Krawczyk-Balska, 2013; Hayakawa et al., 2022). In 1969, the research team of Lowbury et al. isolated a strain of carbencillin-resistant *Pseudomonas aeruginosa* from the blood samples of patients in the United Kingdom, revealing the existence of the IncP-1 plasmid for the first time (Lowbury et al., 1969). The classical IncP-β subtype plasmids, R751 and pB8, were discovered in *Enterobacter aerogenes* in 1974 and *Pseudomonas* sp. in 2000, respectively. These two plasmids have highly similar backbone modules, with differences primarily focused on the accessory modules. R751 contains Tn402, with a structure of *intl1*-*dhfrllc*-*orfD*-*qacE*. In comparison, pB8 carries a transposon derivative of *Tn402*, containing the *bla*_OXA-2_ resistance determinant cluster, with a structure of *intl1*-*bla*_OXA-2_-*aadA4*-*qacE△1*-*sul1*-*orf5*. The sequences on both sides are completely identical to those in R751, suggesting that these two plasmids might have originated from a common ancestor (Schlüter et al., 2005). In *Vibrio alginolyticus*, an IncC plasmid with the *bla*_CMY-2_ resistance gene was found, as well as an IncP plasmid with the *bla*_VIM-1_ resistance gene. The *bla*_VIM-1_ gene was likely acquired by the class I integron and inserted into the integration hotspot of the IncP plasmid, which is different from the IncC plasmid (Zheng et al., 2019). IncP plasmids have been found not only in human samples but also in the natural environment. In 2013, del Castillo et al. identified an IncP-1 plasmid (pP9014) in a strain of *Photobacterium damselae subsp. piscicida* that was sourced from fish for the first time. This plasmid carries genes *tetRD*, *cat*II, and a β-lactam resistance gene. The gene environment structure sequence is as follows: *bla*-IS*5*-*cat*II-*tetR*-*D*-IS*26*-Tn*3 (* del Castillo et al., 2013). In 2021, Yusuke Ota et al. for the first time discovered IncP-1 plasmids harboring the *bla*_KPC-2_ resistance gene in strains of *Citrobacter freundii* (p5_CFTMDU) and *Klebsiella variicola* (p3_KVTMDU). The similarity between the p5_CFTMDU and p3_KVTMDU plasmids reached 99.8%. Both plasmids harbor the *bla*_KPC-2_ resistance gene in the genetic environment of ISKpn*6*-*bla*_KPC-2_-Tn*3* (Ota et al., 2022). These plasmids, including pP9014, p5_CFTMDU, and p3_KVTMDU, highlight the ability of plasmids to serve as carriers for gene transfer between bacteria and natural environments. Moreover, the transposon Tn*3* has become a common genetic element in the dissemination of antibiotic resistance genes.

A total of 316 IncP plasmids have been found in 24 countries, includingChina, Spain, Japan, USA, Viet Nam, Laos, Canada, Czech Republic, France, Germany, Argentina, Brazil, United Kingdom, Belgium, Denmark, Iran, Australia, Croatia, Ghana, Mexico, Missing, Netherlands, and South Korea. These plasmids have been detected in over 60 different host bacteria, with the most frequent one being *Escherichia coli* (n=49 cases) (**Figure 2G**). It is shown that the isolated source of IncP plasmid is relatively evenly distributed in human, environmental and animal samples, but unlike the other plasmids, it is more common as an isolated source in environments such as soil or sewage.

The backbone genes of IncP plasmids include the replication region, stability region, and conjugative transfer region. However, IncP plasmids are the most heterogeneous, and they are not identical to one another. The replication region of IncP plasmids mainly consists of two gene loci: *oriV* (origin of replication) and *trfA* (encoding the protein necessary for *oriV* activation). Genes related to the maintenance region include the par region (*parA, parB, parC, parD*, and *parE*), *korA-kfrA* region, and Tn*1* (present in IncPα plasmids and absent in IncPβ plasmids). Some plasmid backbones contain the *higA-B* toxin-antitoxin system. The conjugative transfer region consists of the *Tra1* and *Tra2* regions. The backbone genes of IncPα and IncPβ plasmids mainly differ in the region between *oriV* and *trfA*, as well as between *Tra1* and *Tra2 (* Pansegrau et al., 1994; Zhao et al., 2017). Antibiotic resistance genes and mobility elements are also present in the accessory modules of IncP plasmids.

Among the β-lactam antibiotic resistance genes carried by IncP plasmids, the *bla*_KPC_ gene has the highest carriage rate, followed by *bla*_OXA_, *bla*_GES_, *bla*_IMP_, and *mcr*. The most common subtypes of *bla*_KPC_ resistance gene are *bla*_KPC-2_, and *bla*_OXA-2_ subtype also commonly appears (**Figure 3**). On IncP plasmids, additional genes for β-lactam antibiotic resistance have also been identified, including *bla*_TEM_, *bla*_VIM_, *bla*_SHV,_ and *bla*_NPS_. Additionally, frequent co-occurrence of *bla*_OXA-48_ and *bla*_IMP-45_ has been observed on IncP plasmids (**Table 2**), potentially forming stable gene cassette sequences (Zhang et al., 2021). Due to the complex gene structure of IncP plasmids, the structure of the carried resistance genes is also quite disordered. No conserved sequences have been found, but some plasmids’ resistance genes are often located downstream of Class I integrons or transposons such as Tn*3*, Tn*402*, or their derivatives. This may indicate the presence of integration hotspots associated with Class I integrons and/or transposons Tn*402*, facilitating the dissemination of resistance genes. IncP plasmids harbor other resistance genes, including *aacA*, *aadA*, *aacC*, *tetAR*, *mer*, *sul1*, *qacEΔ1*, *strAB*, *dhfrllc*, *aac (6’)-lb*, *aac (2’)-IIa*, and *fosA*. They also demonstrate resistance to heavy metals such as Ni, Cu, Zn, and Pb (Yoshii et al., 2015).

Similar to the IncHI plasmid, The IncP plasmid’s conjugation transfer is regulated by the *Tra1* and *Tra2* regions. The *Tra1* region contains core genes such as *traF*, *traG*, *traH*, *traI*, *traJ*, *traK*, *traL*, and *traM*, which are responsible for DNA transfer and replication. The *Tra2* region consists of *trbB*, *trbC*, *trbD*, *trbE*, *trbF*, *trbG*, *trbH*, *trbI*, *trbJ*, *trbK*, and *trbL*, and its main purpose is to facilitate contact between donor and recipient bacteria, as well as the formation of Mpf. *TrbB* and *TrbE* are involved in the pili assembly process, while *traI*, *traH*, *traJ*, and *traK* are involved in DNA processing. *TraM* can increase the frequency of conjugation transfer. Although most IncP plasmids possess conjugation transfer genes, the transfer frequencies vary significantly. It is not possible to analyze the reasons for this difference through the detection of gene sequence variations. This may be attributed to the incompleteness or specificity of the conjugation transfer genes in IncP plasmids (Yano et al., 2013).

Unlike other plasmids, the IncP plasmid has host bacteria that include *Pseudomonas* species, indicating an even broader host range. The IncP plasmid often carries the *bla*_KPC-2_ or *bla*_OXA-2_ genes. Additionally, there have been reports of more structurally complex IncP megaplasmids. These characteristics present significant challenges for clinical anti-infective treatment.

## 6 Conclusions

Incompatible plasmids have appeared in bacteria previously unexplored, along with the continuous enrichment and dissemination of the harbored antibiotic resistance genes, adding a new level to the spread of bacterial resistance genes and becoming the main platform for the transmission of antibiotic resistance genes. The geographical distribution of plasmids also affects the spread of antibiotic-resistant genes. There are certain differences in bacterial populations in different geographical regions, which may be related to human activities, environmental factors, and the use of antibiotics. Each type of plasmid possesses a basic and highly conserved plasmid backbone. Additionally, the high variability of accessory modules carried by plasmids allows for greater diversity among different types of plasmids. These accessory modules contain various genetic elements, which not only carry multiple gene cassettes and resistance genes but also have the ability to capture exogenous genes or provide promoters for resistance genes. The presence of genetic elements provides convenient conditions for the capture, dissemination, and expression of resistance genes, further increasing the rate of resistance formation and dissemination. Different types of incompatible plasmids carry different β-lactamase genes, while resistance genes for sulfonamides and aminoglycosides are also common. With further research, an increasing number of resistance genes associated with plasmids are being discovered. This urgently requires us to conduct more in-depth studies on different genetic elements to establish a comprehensive knowledge system for the enrichment and dissemination of resistance genes, in order to effectively control the spread of multi-drug-resistant bacteria.
